# Structural and Functional Dynamics of the Ovary and Uterus during the Estrous Cycle in Donkeys in the Eastern Caribbean

**DOI:** 10.3390/ani13010074

**Published:** 2022-12-24

**Authors:** Lorenzo G. T. M. Segabinazzi, Robert O. Gilbert, Rachael L. Ambrosia, Don R. Bergfelt, Juan C. Samper, Erik W. Peterson, Hilari M. French

**Affiliations:** Department of Clinical Sciences, Ross University School of Veterinary Medicine, Basseterre P.O. Box 334, Saint Kitts and Nevis

**Keywords:** jenny, follicular wave, estrous cycle, diestrus, artificial insemination

## Abstract

**Simple Summary:**

Donkeys have diverse importance worldwide. Even though donkeys have been replaced by machinery in agriculture and transportation in many parts of the world, in some regions, they continue to be valued for agriculture, transportation, recreation, or even meat or milk production. However, some donkey populations have become feral and represent a nuisance to human populations; therefore, improved contraceptive methods are required. In Brazil and the United States, the exponential growth of donkeys used to produce mules has generated avid interest in donkey reproduction. In addition, some donkey breeds are threatened or endangered, and efforts to save these species depend on enhanced knowledge of reproductive processes. Therefore, for enhancing or inhibiting reproduction, species-specific information is valuable, and understanding the basic mechanisms of reproduction in donkeys is essential for several reasons. While the mare has been extensively studied, few studies have explored follicular dynamics in jennies. Therefore, this study characterized ovarian and uterine dynamics and progesterone profiles in jennies in the Eastern Caribbean. The results of the present study will help in better defining specific differences between breeds of donkeys or donkeys in different areas of the globe, which can be used for either population management or improving reproductive techniques.

**Abstract:**

Eight non-bred, non-pregnant, regularly cycling Caribbean jennies were examined daily via transrectal ultrasound to define the ovarian and uterine dynamics during four consecutive estrous cycles. Blood samples were collected every other day for progesterone analysis. The mean (±SD) overall inter-ovulatory interval across all donkeys and cycles was 22.93 ± 1.99 days. The maximum follicular diameter was 34.6 ± 2.9 mm. A two-wave pattern was evident in 97% (30/31) of the cycles. The emergence of the future dominant follicle and the largest subordinate follicle of the major primary wave coincided on Day 5.7 ± 3.6 post-ovulation, whereas the secondary wave emerged on Day 19.8 ± 2.9 during estrus of the previous cycle or early diestrus. The secondary wave was often minor (93%, 28/30 cycles). Follicular deviation occurred 8.2 ± 1.4 days before the subsequent ovulation. Luteal volume increased for the first four days after ovulation and reached a maximum volume of 8.5 ± 2.7 mm^3^ at Day 5.4 ± 0.4, before gradually regressing after Day 15. Serum progesterone concentration increased from Day 1 after ovulation, peaking at 27.0 ± 9.6 ng/mL between 7 and 10 days after ovulation. Progesterone concentration dropped precipitously around Day 15 after ovulation and was below 2 ng/mL around Day 17 ± 2. A day effect (*p* < 0.0001) was observed for corpus luteum’s volume, progesterone concentration, and uterine tone, but not for endometrial edema (*p* > 0.05). This study helps to clarify and define normal estrous characteristics of jennies in the Eastern Caribbean.

## 1. Introduction

Historically, donkeys have been used by humans for transportation and agricultural work, but with the development of machinery in agriculture and transportation, donkeys have been replaced in most areas of the globe. However, in so-called developing areas, the importance of donkeys remains high [1]. Fortunately, the exponential growth of donkey breeds used for shows and to produce mules (e.g., the Mammoth donkey in the USA and the Pêga donkey in Brazil) and the increased demand for donkey milk in the cosmetic industry and for hypoallergenic consumption has generated avid interest in donkey reproduction [2,3,4]. In addition, some donkey breeds are threatened with extinction, due to loss of breeding animals (e.g., Catalan, Baudet du Poitou, Miranda, and Ragusano in Europe). In contrast, others became feral and represented a nuisance overpopulation of unwanted donkeys in some regions (e.g., desert areas of the United States, some Caribbean Islands, and northeast Brazil) [5,6]. To enhance or inhibit reproduction, species-specific information is a fundamental requirement [7].

Among the reproductive organs, the ovaries are one of the most dynamic systems responsible for regulating critical reproductive events in the female reproductive cycle. Follicular growth, development, and ovulation, as well as luteal growth, development, and regression, are some of the important events in the ovaries that affect other reproductive organs, such as the uterus and the cervix. While information regarding follicular wave dynamics, ovulation, and structural and functional aspects of corpora lutea maturation and regression have been extensively studied in horse and pony mares [8,9,10,11,12,13,14], this information is far less known for donkeys [15,16,17,18,19,20]. Moreover, there is contradicting information regarding the donkeys’ estrous cycle [15,16,20]. For example, inter-ovulatory intervals and follicular wave patterns for the Martina Franca donkey in France [15] and the Dezhou Black in China [17] varied significantly from data reported about jennies in upper Egypt [16] and Mexico [18]. In addition, most, if not all, studies describing the donkeys’ estrous cycles were performed in sub-tropical and temperate zones [5,15]. Hence, there is a lack of information about jennies’ estrous cycles under tropical conditions in the Eastern Caribbean [20]. Understanding species-specific structural and functional dynamics of the ovary and the uterus is essential for formulating hypotheses, diagnoses, and prognoses of reproductive function. The present study is aimed at characterizing follicular wave development, structural and functional maturation and regression of the corpus luteum (CL), and uterine dynamics during the estrous cycle in jennies in the Eastern Caribbean.

## 2. Materials and Methods

### 2.1. Animals and Animal Management

This study was reviewed and approved by the Ross University School of Veterinary Medicine, Institutional Animal Care and Use Committee (RUSVM, IACUC 11.16.32) and conducted from February to August 2017 at RUSVM, Basseterre, St. Kitts, West Indies (17°18′ N 62°44′ W) in the Eastern Caribbean. A total of eight non-bred, non-pregnant Caribbean jennies 3.5–10 yrs of age (mean ± SD, 6.0 ± 3.0 yrs), weighing 95–174 kg (142.5 ± 18.4 Kg), and measuring 95.5–108 cm high at withers (104.7 ± 6.3 cm) were used for the study. Jennies were selected based on a good body condition score (5 on a 7-point scale [21]), the absence of clinical disorders, regular cyclicity confirmed by observed signs of estrus, and ultrasonographic imaging of a CL during diestrus. The animals were housed in outdoor grass paddocks under natural light, fed with freshly cut New Guinea grass (*Megathyrsus maximus*), and had free access to water and shade.

### 2.2. Study Design

The experiment was conducted from Day 0 (ovulation = Day 0) of the first estrous cycle until Day 0 of the beginning of the fifth estrous cycle, thus totaling four complete cycles or inter-ovulatory intervals (IOI) for each of the eight jennies. The jennies were monitored daily by transrectal ultrasonographic imaging (5 mHz probe, Sonosite, Universal) to detect, measure, and map antral follicles ≥2 mm, detect ovulation, and monitor CL development. During each ultrasound examination, follicles and CL in each ovary were identified and recorded. Cross-sectional measurements of the antrum of follicles ≥2 mm and CL were obtained using internal calipers of the ultrasound machine.

### 2.3. Follicles and Follicular Waves

Follicle diameters were classified as small (5 to 10 mm), medium (11 to 19 mm), and large (≥20 mm). To characterize follicular waves, all follicles ≥2 mm were tracked and recorded daily using other follicles’ CL as references in the daily tracking of each structure. Follicle tracking started with a diameter ≥2 mm. The day of emergence of a follicle was defined as the day before the follicle first exceeded 6 mm [10]. Follicles that emerged ≤2 d apart were considered a cohort of the same follicular wave, whereas those that emerged >2 d apart were considered a cohort of follicles of a different wave [22,23]. The type, number, and frequency of follicular waves were characterized as major or minor and primary or secondary [10]. Major waves gave rise to a dominant follicle (DF) that regressed, formed a hemorrhagic anovulatory follicle (HAF), or ovulated. Minor waves were characterized as the absence of a DF. Primary major waves emerged during diestrus and gave rise to the ovulatory DF, whereas secondary minor or major waves emerged during late estrus of the previous cycle or early diestrus of the extant cycle. Follicle deviation was defined as the first day of progressively increasing difference between the largest or future DF and the largest or future subordinate follicle (LSF) [11]. Follicle growth rates were characterized as the difference between the minimum and maximum diameter of the follicle divided by the time from emergence to the start of regression or ovulation.

### 2.4. Ovulation and CL Structure and Function

Once a follicle grew to ≥30 mm as a DF, ultrasonic imaging was done every 6 h to more accurately determine preovulatory size and time of ovulation. The day of ovulation was regarded as the day on which the DF was no longer observed. The IOI was characterized as the time between one ovulation and the next ovulation.

To characterize structural maturation and regression of the CL, volume was calculated based, in part, on the longest dimension and the widest perpendicular dimension of the CL and the central anechoic cavity, if present. Volume was calculated according to the formula of an ellipsoid, 4/3.*π.a.b.c*, where a = half of the longest dimension and b = c = the radius of the perpendicular dimension of the CL [24]. For a CL with a central cavity, volume was calculated after subtraction of the volume of the central cavity.

To characterize functional maturation and regression of the CL, blood samples were collected every other day throughout the estrous cycles for progesterone analysis. Samples were collected by venipuncture of the external jugular vein using an 18 G needle and tubes without anticoagulant. After collection, the blood was allowed to clot at room temperature (~22 °C) for 6 h, and serum was aspirated and allocated into labeled microcentrifuge tubes and stored at −80 °C in a freezer until progesterone analysis. Progesterone concentrations were assessed using a commercial enzyme immunoassay kit (Arbor Assay, K025-H5; Ann Arbor, MI, USA) that was previously validated for use in donkeys [25]. Briefly, serum samples were run in duplicate using 50 µL of sample diluted 1:16 with assay buffer. Extrapolation of progesterone concentrations that fell outside the range of the reference standard curve were further diluted and re-analyzed so that concentrations fell within the range of the standard curve for increased accuracy. Within-assay CV and sensitivity were 19% and 0.04 ng/mL, respectively. Structural and functional luteolysis was considered when the volume of the CL declined by 50% and progesterone concentrations declined by 50%. Luteolysis was considered complete when progesterone concentrations decreased to less than 2 ng/mL [26]. 

### 2.5. Uterine Dynamics

Daily transrectal digital compression of the uterine horns and cervix was carried out to characterize uterine tone (1 to 3; 1, flaccid and 3, turgid) [27]. Correspondingly, transrectal ultrasonographic imaging was carried out to characterize endometrial edema of the uterus based on cross-sectional images of both uterine horns and the longitudinal image of the uterine body (1, minimal edema to 4, exacerbated edema) [27,28].

### 2.6. Statistical Analysis

Data analyses were carried out with GraphPad Prism 9.0.1. (GraphPad Prism 9.0.1., GraphPad Software, San Diego, CA, USA). The number of follicles by class, diameters and growth rates of DF and LSF, interovulatory intervals, the volume and growth rates of the CL, uterine tone, and endometrial edema scores were compared among cycles and jennies and within each cycle (day effect). The normality of the data was evaluated by a Shapiro–Wilk normality test. Interovulatory intervals, the number of follicles by class, diameters, and growth rates of DF and LSF, and the volume and growth rates of the CL were assessed using a mixed model with Tukey’s post hoc test to identify the mean differences among days of the estrous cycle. Uterine tone and endometrial edema scores were tested using the Kruskal–Wallis test, followed by Dunn’s test. Data are summarized as mean ± SD unless otherwise stated. Repeatability was calculated as the intraclass correlation coefficient (ICC). Significance was set at *p* ≤ 0.05 for all tests. The degree of linear correlation between preovulatory follicle diameter, CL volume, progesterone concentrations, uterine tone, and endometrial edema was tested using Pearson’s coefficient correlation. A strong coefficient of correlation was defined as r > 0.7; a moderate coefficient of correlation was defined as 0.5 > r < 0.7; a weak coefficient of correlation was defined as r < 0.5.

## 3. Results

In total, 31 estrous cycles (4 cycles/8 jennies) were evaluated. One cycle was excluded due to the development of a hemorrhagic anovulatory follicle (HAF). The jenny subsequently ovulated 19 d later and presented normal cyclicity afterward. Among the jennies, ovulations occurred equally (*p* > 0.05) from the left (52%, 16/31) and right (48%, 15/31) ovaries. Mean IOI across all donkeys and cycles was 22.93 d (±1.99 d; range 20–25 d) and repeatable within each jenny (ICC–0.46; *p* < 0.05) but different between jennies (*p* = 0.004). Longer IOIs were associated with larger follicles and longer follicular phase (r = 0.58; *p* = 0.0008 and r = 0.54; *p* = 0.0023, respectively). 

**Figure 1:**
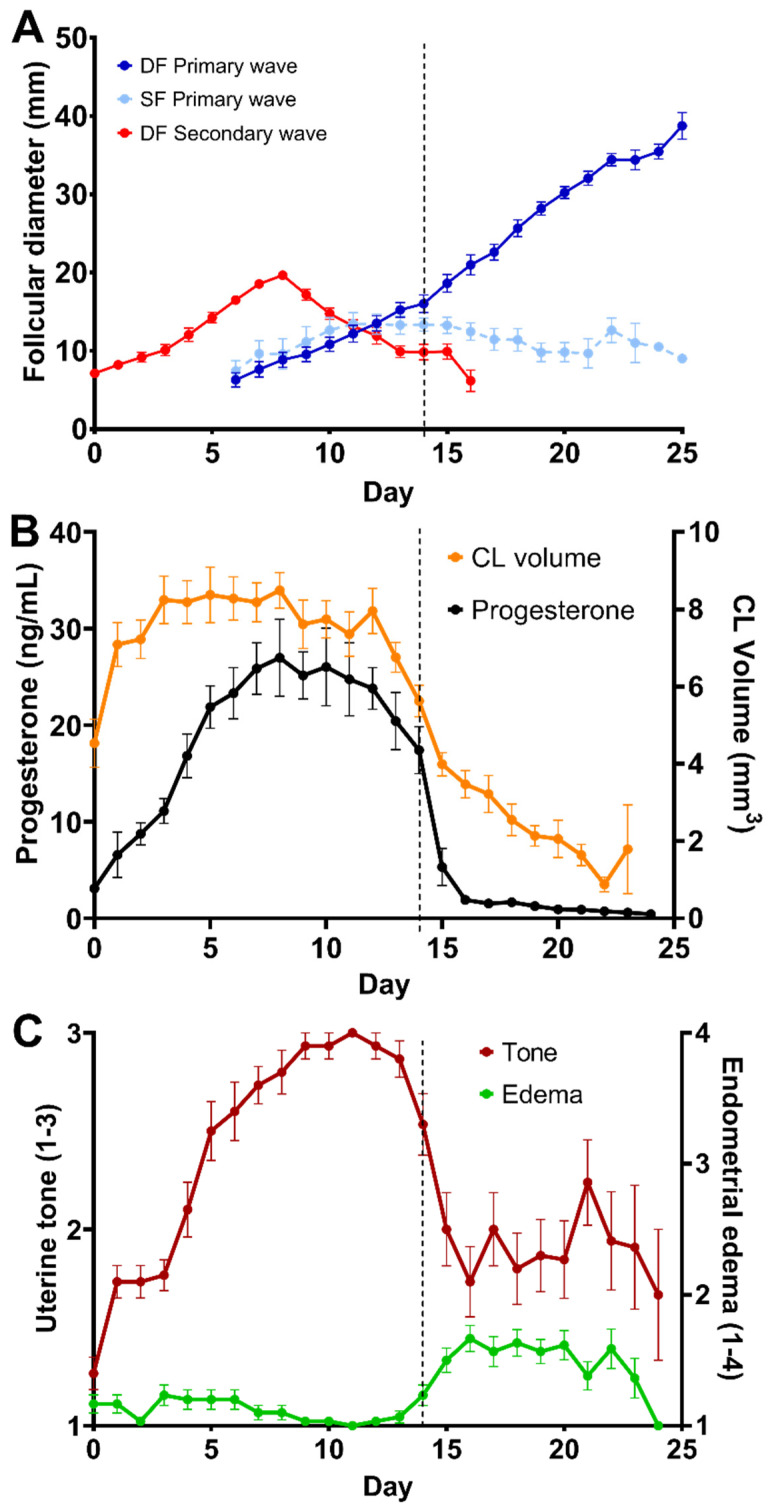
Mean ± SEM of (**A**) follicular growth in the primary and secondary waves, (**B**) endometrial edema and uterine tone scores, and (**C**) volume of corpus luteum and serum progesterone concentrations in four estrous cycles of Caribbean jennies (n = 8). Dashed line indicates the follicular divergency. The CL volume was calculated according to the formula for the volume of an ellipsoid, 4/3.*π.a.b.c*, where a = half of the longest dimension and b = c = the radius of the ‘waist’ or widest part of the CL. Uterine tone (1 to 3; 1: flaccid and 3: turgid) and endometrial edema (1: minimal edema to 4: exacerbated edema) scores [28] were evaluated by transrectal digital palpation and ultrasonography, respectively.

**Figure 2:**
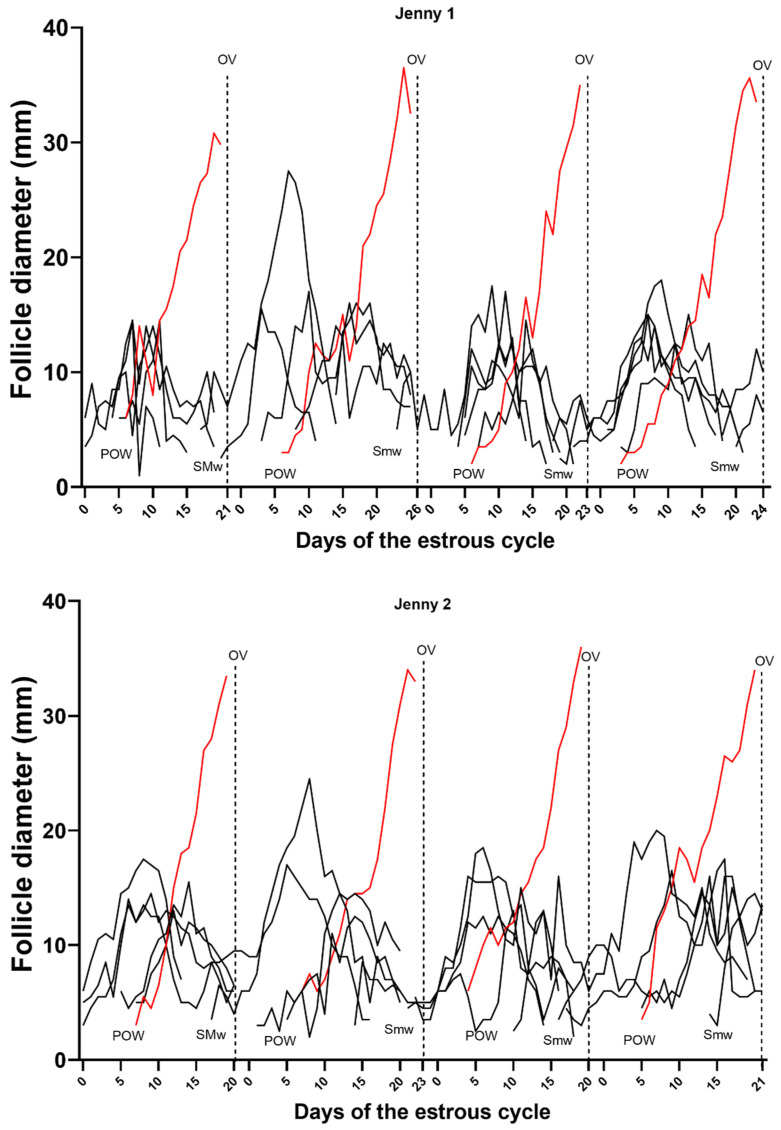
Profile of individually tracked follicles of two selected Caribbean jennies during four inter-ovulatory intervals (IOIs). The types of follicular waves characterized by inspection of the follicle data (with identity) are shown during each IOI for each jenny. The red line represents the dominant follicle of each wave. POW, primary ovulatory wave; SMw, major secondary wave; Smw, minor secondary wave; OV, ovulation.

Follicles and follicular waves and structural and functional characteristics of the CL are shown in Table 1. A two-wave pattern was observed in 97% (30/31) of the cycles, whereas one estrous cycle throughout the study (3%, 1/31) had a one-wave pattern. Emergence of future DF and LSF of primary waves occurred simultaneously on Day 5.7 ± 3.6 post-ovulation, with similar growth rates at 1.4 ± 0.7 mm/day until deviation, which occurred on Day 14.1 ± 1.5, or 8.2 ± 1.4 days before ovulation (Figure 1A). The diameter of the DF at deviation was 14.5 ± 2.6 mm, with a growth rate from deviation to ovulation of 2.6 ± 0.7 mm. After deviation, the LSF plateaued for two days with a growth rate of 0.2 ± 0.1 mm/day and, thereafter, decreased in diameter (*p* < 0.05). The mean follicular growth rate during the estrous cycle was 1.9 ± 0.52 mm per day. While the mean maximum diameter of the DF before ovulation was 34.64 mm (±2.91 mm, range 30–43 mm), there was a reduction in size and shape change as ovulation approached (34.11 ± 3.31 mm; *p* < 0.05). The secondary wave was often a minor wave (93%, 28/30 cycles), as it did not give rise to a DF. Secondary waves emerged on Day 19.8 ± 2.9 (Figure 1A and Figure 2) post-ovulation of the preceding cycle, with the largest follicle reaching the maximum diameter (19.7 ± 1.8 mm) around day 7.4 ± 1.2 post-ovulation of the extant cycle. While a majority of the secondary waves were minor waves (93%, 28/30 cycles), major secondary waves were observed in two jennies with a DF (7%, 2/30 cycles) but no diestrus ovulations (Figure 2). The follicular growth of each of the jennies used for the study during four estrous cycles is depicted in Figure S1.

The total number of antral follicles > 2 mm was consistent within jennies and between ovaries within jennies (ICC = 0.72; *p* < 0.0001), but different between jennies (controlling for the day; *p* < 0.0001). Mean numbers of small-, medium- and large-sized follicles were similar among days of the estrous cycle (*p* > 0.05; Figure S2; Table 1). Interestingly, there was a small but significant negative correlation between the total number of follicles present throughout a cycle and serum progesterone concentration (r = −0.20; *p* = 0.0009).

A day effect (*p* < 0.0001) was observed for CL volume and serum progesterone concentration. The corpus luteum volume increased four days after ovulation and reached a maximum volume (8.5 ± 2.7 mm^3^) at Day 5.4 ± 0.4, before gradually decreasing after Day 15 (Figure 1B). The volume of the CL was correlated with the diameter of the DF for 9 d post-ovulation (r = 0.62, *p* < 0.001). Serum progesterone concentration increased after ovulation, reaching maximum concentrations of 27.0 ± 9.6 ng/mL between 7–10 d (7.8 ± 0.8) and, thereafter, decreased, reaching low concentrations by Day 15 (Figure 1B, Table 1) and <2 ng/mL on Day 17 ± 2. The mean progesterone concentration and the luteal volume were correlated (r = 0.61; *p* < 0.05) throughout the estrus cycle. As previously defined, the mean (±SD) Day of luteolysis across all cycles and donkeys was 15.2 ±1.7 d and was repeatable within jennies (ICC–0.52; *p* < 0.05) and between jennies (*p* > 0.05). There was a day effect (*p* < 0.0001) for uterine tone, but not for endometrial edema (*p* > 0.05; Figure 1C). Uterine tone increased four days after ovulation, peaked around Day 9.5 ± 1.8, and decreased at Day 15.0 ± 1.5 (*p* < 0.05; Figure 1C). The uterine tone was negatively correlated with the development of the DF of the primary follicular wave (r = −0.64; *p* < 0.05) and positively correlated with CL volume (r = 0.59; *p* < 0.05) and progesterone concentrations (r = 0.76; *p* < 0.05). Endometrial edema remained low after ovulation for about 14–15 days. Although a slight increase was observed between Day 16.0 ± 1.0 until 1.2 ± 1.1 days before ovulation, it was not different compared to the rest of the estrous cycle (*p* > 0.05; Figure 1C). The endometrial edema pattern was repeatable within jennies (ICC–0.51; *p* < 0.05), but different between jennies (*p* < 0.001). Endometrial edema was positively correlated with the development of the DF of the primary follicular wave (r = 0.32; *p* < 0.05) but negatively correlated with progesterone concentrations (r =−0.58; *p* < 0.05). A box plot of uterine tone and endometrial edema scores is highlighted in Figure S3.

***Periovulatory period temporal relationships***

**Figure 3:**
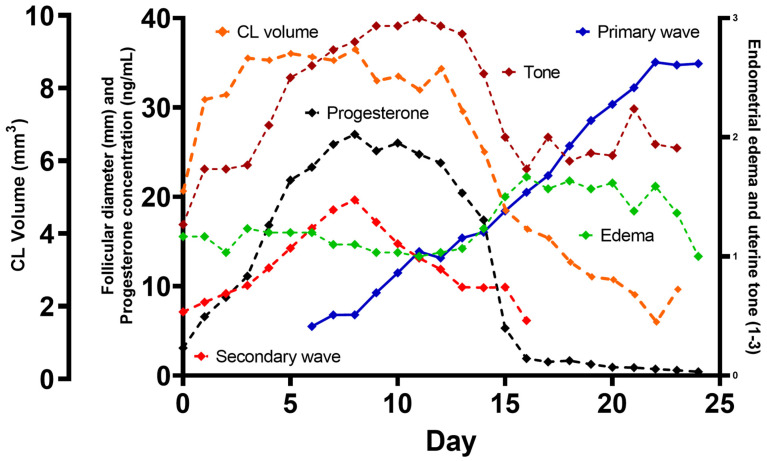
Mean temporal relationships among the diameter of the dominant follicle in the primary and secondary follicular waves, CL volume, progesterone concentrations, and mean scores of the uterine tones and endometrial edema during four estrous cycles of eight Caribbean jennies (n = 31) under tropical conditions. Day 0, day of ovulation.

The temporal relationships among the diameter of the DF in the primary and secondary waves, CL volume, progesterone concentrations, and mean scores of the uterine tones and endometrial edema are highlighted in Figure 3. After ovulation, the uterine tone score increased (*p* < 0.05) following the development of the CL and the consequent increment in progesterone concentration. At the same time, although not statistically significant, the endometrial edema score slightly decreased (*p* > 0.05) and remained low until the appearance of the preovulatory follicle in the primary follicular wave and a significant reduction in CL volume and progesterone concentrations. As ovulation approached, the growth rate of the preovulatory follicle decreased until ovulation (*p* < 0.05).

## 4. Discussion

The results of the present study revealed several ovarian and uterine characteristics that appear to be specific for Caribbean jennies and other features similar to small-frame donkey breeds. Characteristics such as length of the estrous cycle, follicle diameter at ovulation, and incidence of multiple ovulations have been reported to be influenced by the breed in donkeys [5,17,19,20,29,30,31,32,33] and horses [11,34,35,36]. The duration of the IOI in Caribbean jennies appeared to be slightly shorter (22.9 days; range: 20–25 days) than observed in other donkey breeds, such as Mammoth, Catalonian, Pega, Standard, Mexican Burro, and crossbreed donkeys (23.3–24.9 days; range: 23–30 days) [5,17,18,19,29,30,32]. The reasons for the shorter IOI in Caribbean jennies are unknown but could be associated with the photoperiod in this tropical region, as observed in thoroughbred mares in a tropical environment [37]. Interestingly, as similarly reported in Mexican Burro jennies [18], longer IOIs were associated with longer follicular phases in Caribbean jennies, whereas a relatively constant luteal phase was observed throughout the study. The reasonably regular length of the luteal phase has also been reported in mares [37,38] and women [39,40], with variations in cycle length associated with differences in the follicular phase. 

Only one anovulatory cycle was observed during the present study. The present study was performed during the breeding season in the northern hemisphere, and although the reproductive activity of jennies is less affected than that of mares by seasons [38], some authors have reported differences in ovulation rates in jennies around the year [33,39,40]. Of interest, the formation of HAF has been reported in 4.6% of the cycles of Dezhou Black jennies, with no difference between seasons [17]. Single ovulations characterized all of the remaining cycles in the present study, as reported in Mexican Burro jennies in one study [18]. However, previous studies reported a variation between 5 and 32% of multiple ovulations in other donkey breeds [5,17,21,29,32,40]. It is worth noting that Caribbean and Mexican Burro donkeys share similarities regarding body weight (100–140 kg) and are smaller than most donkey breeds used in previous studies, which might be a factor associated with the low incidence of multiple ovulations in these donkeys. Low incidence (5%) of multiple ovulations has also been described in Brazilian Northeastern jennies, another small-frame donkey breed [21], as well as in miniature horses [41]. 

A considerable variation in preovulatory follicular diameters (30–43 mm) was observed in Caribbean jennies in the present study. Similar variation (32–45 mm) was also reported in Brazilian Northeastern jennies [21] and other donkey breeds [17,29,32,42]. The diameter of the preovulatory follicle in Caribbean jennies (34.6 mm) in this study was similar to that reported for other donkey breeds, such as Amiata, Brazilian native, Pega, Andalusian, Mexican Burro, and Zamorano–Leones donkeys [18,32,43,44,45], but smaller than the preovulatory follicular size of larger donkey breeds (e.g., Martina Franca [43.1 ± 0.4 mm] [15], Miranda [40.2 ± 1.4 mm] [46], and Dezhou Black jennies [39.5 ± 2.6 mm] [17]). The smaller diameter of the preovulatory follicle in some donkey breeds may be associated with lower body weight, which has also been described between miniature ponies and large horses, although this difference (preovulatory follicle diameter) is small when the relative difference in body weight is considered [41]. Moreover, although there was considerable variation in the diameter of the preovulatory size among the jennies in the present study, the repeatability of the diameter of the preovulatory follicle between estrous cycles of the same jenny was similar to that previously reported in mares [34,47,48] and donkeys [15,17,18,32]. In addition, the reduction in follicular growth and changes in follicular shape before ovulation observed in Caribbean jennies and also reported in other donkey breeds [16,17,18] and mares [8,36,47,49,50] can help to predict the moment of ovulation, which can assist the reproductive technology in this species, as has been done in mares [35,51,52,53]. Knowledge about the repeatability of the diameter of the preovulatory follicle and morphological changes in the follicle before ovulation can be beneficial in managing different reproductive goals in breeding programs. Determination of the diameter of the preovulatory follicle and its growth pattern during multiple estrous cycles provides knowledge for improving reproductive management practices in jennies in the Caribbean tropics.

Through the studied estrous cycles, two follicular waves were observed in most of the estrous cycles in Caribbean jennies. A primary wave, which gave rise to the dominant and ovulatory follicle, originated during diestrus (Day 5.7 ± 3.6) and was observed in all the studied cycles in the Caribbean jennies in the present study. The second wave was characterized by emergence during the late estrus or early diestrus with no development of a dominant follicle (minor wave) in 93% of the cycles and divergence and development of a dominant follicle (major wave) but no ovulation in 7% of the cycles in the present study, as previously described in mares [8,9,10,14,41,54,55]. Few studies have reported the wave pattern in donkey species, with varied results [16,17,18,32]. While a one-wave pattern was recorded in native Egyptian jennies, with the emergence of the ovulatory follicle during estrus of the previous cycle [16], two waves were reported in 48% of the estrous cycles in Mexican Burro jennies; however, the emergence of the follicles was not reported in the study [18]. The authors noted that the second-largest follicle never grew to >20 mm in diameter in Mexican Burro jennies [18], which was similar to what was observed in 93% of the cycles in Caribbean jennies; this might be a characteristic of these donkey breeds and may explain the lack of multiple ovulations. Two to three follicular waves have been described in other larger donkey breeds, such as Pega [31] and Dezhou Black jennies [17]. Interestingly, the three follicular wave patterns observed in Dezhou Black jennies [17] occurred in autumn. In contrast, two follicular waves, similar to those of the Caribbean jennies in the present study, were observed during the rest of the year in Dezhou Black jennies. The authors suggested that the three-waves pattern in Dezhou Black jennies was associated with changes in FSH surges observed during autumn [17], which has also been described in mares [56] and might be associated with some degree of seasonality in donkey species, although the reproductive activity of jennies has been shown to be less affected by seasons than that of mares [17,31,33]. It is worth noting that the present study was performed during the breeding season in the northern hemisphere. Even though there is a lack of difference between seasons in the tropical region (Eastern Caribbean), the authors speculate that similar reproductive patterns may happen in Caribbean jennies in autumn; however, this needs further investigation.

In the present study, the number of follicles per day in the ovary in Caribbean jennies was similar to those reported in Pega donkeys [32] and mares [13,47]. The population of small follicles (≥5 ≤ 10 mm diameter) seems to be constantly present in the ovary, which suggests action as a reservoir to the follicular waves [56]. The follicle emergence and deviation of the DF and SF in Caribbean jennies occurred similarly to those described in Dezhou Black jennies [17] but different from those of native Egyptian jennies [16]. In Caribbean and Dezhou Black jennies, the secondary or anovulatory wave was raised during the previous estrus or the early diestrus, whereas the primary wave or ovulatory wave appeared during early diestrus (Caribbean, Day 5.7 ± 3.6 days; Dezhou Black, Day 8.3 ± 2.1). The follicular deviation occurred at Days 14.1 ± 1.5 and 15 ± 1.2 in Caribbean and Dezhou Black jennies, respectively [17]. In native Egyptian jennies, the emergence of the ovulatory follicle occurred earlier (before ovulation), as did the divergence (Day 8.0 ± 0.84); however, it did not reduce the IOI compared with other donkey breeds [5,17,18,19,29,30,32]. When three waves were developed during the estrous cycle in Dezhou Black jennies, the ovulatory wave and deviation occurred later during diestrus (Day 12.7 ± 2.5 and 16.8 ± 1.1), and the length of the estrous cycle was increased [17]. 

Even though the day of follicle deviation in Caribbean jennies in the present study occurred in a similar timeline as that reported in Dezhou Black jennies, the follicular diameter during deviation was smaller (14.5 ± 2.6 mm) in the present study compared to those of Dezhou Black jennies (19.3 ± 2.0 mm) [17], native Egyptian (22.5 mm), and jennies in Ethiopia (17.9 ± 4.6 mm). Interestingly, the follicle diameter during deviation did not change in Dezhou Black jennies presenting three follicular waves during the estrous cycle, which may explain the longer IOI observed in the study [17]. Although follicular diameter during deviation and ovulation were smaller in Caribbean jennies, the growth rate of the DF after divergence was similar (2.6 ± 0.7 mm/day) to that reported in other donkey breeds [16,17,57]. In this regard, a reduction in the growth rate of the preovulatory follicle between the maximum diameter and ovulation, as described in other donkey breeds [16,17,18], in miniature ponies [41], and in horses [8,36,47,49,50], was also observed in the jennies in this study. In addition, the interval from deviation to ovulation was similar for the jennies in this study (8.2 ± 1.4 days) and Dezhou Black jennies (8.3 days), [17] but shorter compared to that of native jennies in Egypt (9.6 days) [16]. 

The CL development and regression in Caribbean jennies followed similar patterns as previously described in other donkey breeds [15,16,18,32]. The present study observed a moderate correlation between preovulatory follicle diameter and CL volume in the Caribbean jennies. A similar correlation was described in mares [58] and jennies in another study by our group [25]. The CL reached the maximum volume around Day 5 after ovulation and decreased slowly until Day 11, then declined precipitously along with a decline in serum progesterone concentrations [15,32]. The progesterone profile in Caribbean jennies during the estrous cycle was similar to that reported in Dezhou Black, Mexican Burro, and French jennies [17,18,57]. Progesterone in the jennies was <2.0 ng/mL in the presence of a preovulatory follicle and >2 ng/mL when a CL was present. After ovulation, the progesterone concentrations increased gradually until they reached a plateau between Days 7 and 10 post-ovulation and decreased precipitously around Day 15 to ≤1 ng/mL three to four days later. The timing of the abrupt drop in progesterone concentration in this study’s jennies differs slightly from the observations in mares [26] and jennies [32], for which a steep decline in concentration was reported between Days 14 and 16. A similar pattern of luteolysis was observed in our previous study with Caribbean jennies [25] and by others with respect to Mexican Burro jennies [18]. The mean time of the onset of luteolysis (Day 15) in the present study was similar to that reported in Mexican Burro jennies [18] and mares [26]. Completion of luteolysis based on ≤2 ng/mL of progesterone occurred later in the present study’s jennies (Day 18–19) than in mares presenting a similar luteolytic pattern [26], but it was similar to that of Mexican Burro jennies [18]. The longer time required for progesterone concentrations to fall below 2 ng/mL in this study’s jennies might be associated with circulating progesterone concentrations during the luteal phase that were higher than those reported in mares [26,58,59,60]. Consistent with knowledge of luteolysis in mares,, the decline in CL volume was not as dramatic as the reduction in progesterone concentrations. In contrast with what is described in mares, although the size of the CL decreases significantly in jennies after luteolysis, the CL does not promptly disappear, and the CL can be detected until the next ovulation [16,32]. 

Morphological changes in uterine tone are used clinically and experimentally to determine the phase of the estrous cycle. They are attributed to alterations in circulating concentrations of progesterone and estradiol concentrations during the estrous cycle. The present results indicated higher uterine tone in Caribbean jennies from ovulation until around Day 14 of the estrous cycle (diestrus period). After that, uterine tone decreased until three days after ovulation, when it started to increase again. Similar uterine tone dynamics during the estrous cycle have been described in mares [27,61].

Conversely, low endometrial edema characterized the diestrus period in Caribbean jennies, slightly increasing during the estrus period. The presence of endometrial edema is a consequence of high estradiol concentrations in the bloodstream secreted by the preovulatory follicle [62,63,64], and although concentrations of estradiol are increased during the follicular phase in Dezhou Black and Martina Franca jennies [15,17], it has not been associated with the endometrial edema pattern in donkey species [21]. High estrogen levels during estrus stimulate the preovulatory secretion of GnRH and an LH surge [65], culminating in the ovulation of a mature follicle [66]. Therefore, the definition of the exact moment of the beginning of the estrous phase is a meaningful finding to facilitate reproductive management. The dynamic of the endometrial echotextural changes during the estrous cycle has been well-documented in horse mares [36,41,67,68]. In mares, this pattern is characterized by pronounced edema during early estrus, with a peak one to two days before ovulation, which decreases 24 h before spontaneous ovulation [67,69]. Although changes in endometrial echotexture were observed between the estrus and diestrus periods in Caribbean jennies, these changes were not as pronounced as they were in mares. Data are insufficient to confirm the inconsistency of endometrial edema scores in relation to the estrus cycle phase in donkey species. However, similar results have been described in Brazilian Northeastern jennies [21], reinforcing the hypothesis that jennies do not have an edema pattern associated with the closeness to ovulation. 

It is important to note that a limited number of jennies was used in the present study, and repeated examinations were performed throughout the study. If more animals had been evaluated, different results could have been obtained. However, the authors feel confident that the results expressed in this study are representative of the structural and functional dynamics of the ovary and uterus during the estrous cycle in donkeys in Eastern Caribbean, as the number of animals used was similar to the numbers reported in several studies (range 6–13) in donkey species [15,16,17,18,20,21,30,32,33,57,70]. In addition, studies have shown that reproductive transrectal palpation and ultrasonography examinations performed both by experienced veterinarians or students do not elicit a major physiological or behavioral stress response in mares [71,72,73]. Similar observations have been made by our group in donkeys [74,75,76,77].

## 5. Conclusions

This study has comprehensively shown the ovarian and uterine dynamics in jennies in the Eastern Caribbean during four consecutive IOIs during the reproductive season. The mean IOI length was 22.9 days, and ovulation occurred when preovulatory follicles reached 34.6 mm in diameter. Luteolysis occurred around 15 days post-ovulation and was associated with a physiologically steep drop in progesterone concentrations and morphological regression of the CL. Interestingly, the CL was visible until the next ovulation as a corpus albicans (increased echotexture). The uterine tone was associated with the stage of major follicular wave development and CL maturation and regression during the estrous cycle, but endometrial edema did not statistically change between the luteal and follicular phases in jennies in the present study. The present study’s findings are of fundamental importance for a better understanding of ovarian and uterine physiological patterns during the estrous cycle in jennies in the Eastern Caribbean.

## Figures and Tables

**Table 1:** Characteristics of follicular waves and structural and functional development of the corpus luteum of four estrous cycles in eight jennies.

Characteristic	Mean ± SD
***Interval (days) from***	
Length of IOI	22.9 ± 2.0
Ovulation to follicular wave emergence	
Primary waves	5.7 ± 3.6
Secondary waves	19.8 ± 2.9
Ovulation to follicle deviation	14.1 ± 2.9
Primary wave emergence to deviation	8.4 ± 2.1
Follicle deviation to ovulation	8.2 ± 1.4
***Follicle diameters (mm)***	
Dominant follicle at deviation	14.5 ± 2.6
Largest subordinate follicle at deviation	13.3 ± 1.8
Dominant follicle maximum diameter prior to ovulation	34.6 ± 2.9
***Follicle growth rate (mm/day)***	
Emergence to deviation	
Dominant follicle	1.5 ± 0.7
Largest subordinate follicle	1.6 ± 0.6
Deviation to ovulation	
Dominant follicle	2.6 ± 0.7
***Number of follicles per day***	
Small (≥5 to ≤10 mm)	8.3 ± 1.1
Medium (11 to 19 mm)	3.5 ± 1.0
Large (≥20 mm)	0.6 ± 0.5
Total	12.5 ± 1.7
***Corpus luteum***	
Day of maximum size after ovulation	5.4 ± 0.4
Maximum size (mm^3^)	8.5 ± 2.7
Day of maximum progesterone concentration	7.8 ± 0.8
Maximum progesterone concentration (ng/mL)	27.0 ± 9.6

Follicular wave emergence is the day the largest follicle was ≥6 mm in diameter. Follicle divergence was the first day of progressively increasing difference in diameter between the largest and the second-largest follicles.

## Data Availability

The original contributions presented in the study are included in the article/Supplementary Materials; further inquiries can be directed to the corresponding author/s.

## Supplementary Materials

The following supporting information can be downloaded at: https://www.mdpi.com/article/10.3390/ani13010074/s1, Figure S1. Individual development of the largest follicles in the Primary and Secondary waves in four estrous cycles of eight Caribbean jennies in the tropics. HAF, hemorrhagic anovulatory follicle; Figure S2. Number of small (≥5 ≤ 10 mm diameter); medium (11–19 mm diameter) and large (≥20 mm diameter) follicles during four estrous cycles of eight Caribbean jennies in the tropics; Figure S3. Box plot of (A) endometrial edema (B) and uterine tone scores of eight Caribbean jennies during four consecutive estrous cycles in the tropics. Uterine tone (1 to 3; 1: flaccid and 3: turgid) and endometrial edema (1: minimal edema to 4: exacerbated edema) scores were evaluated by transrectal digital palpation and ultrasonography, respectively.
