# Supplementary material for: Structural and Functional Dynamics of the Ovary and Uterus during the Estrous Cycle in Donkeys in the Eastern Caribbean
Source: Animals (Basel). 2022 Dec 24;13(1):74. doi: 10.3390/ani13010074 (PMC9817998; doi:10.3390/ani13010074)
Supplement: Supplementary file 1 [file animals-13-00074-s001.zip › animals-2087780-supplementary.pdf]

Supplement

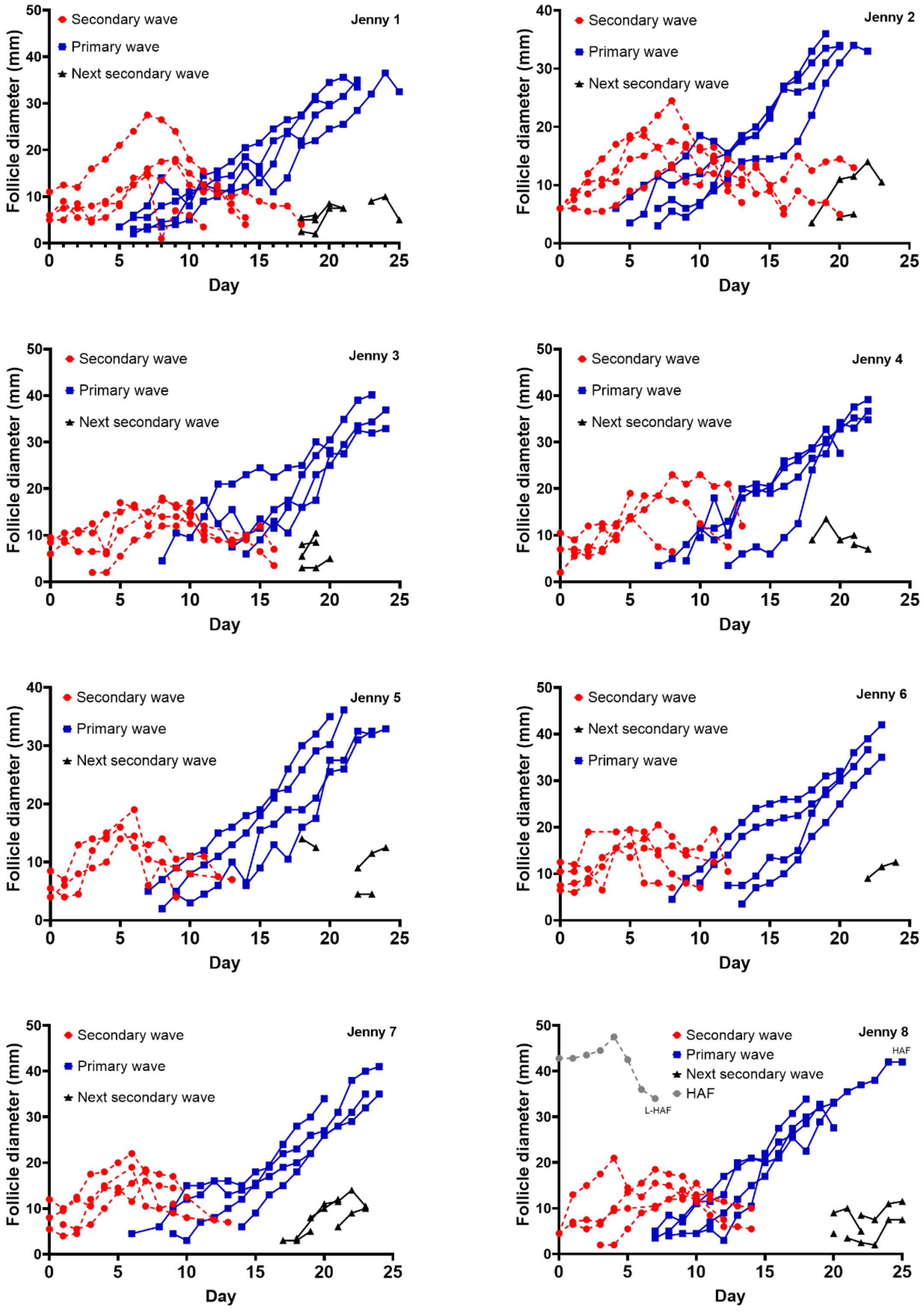

Figure S1. Individual development of the largest follicles in the Primary and Secondary waves in four estrous cycles of eight Caribbean jennies in the tropics. HAF, hemorrhagic anovulatory follicle. L-HAF, luteinized hemorrhagic anovulatory follicle

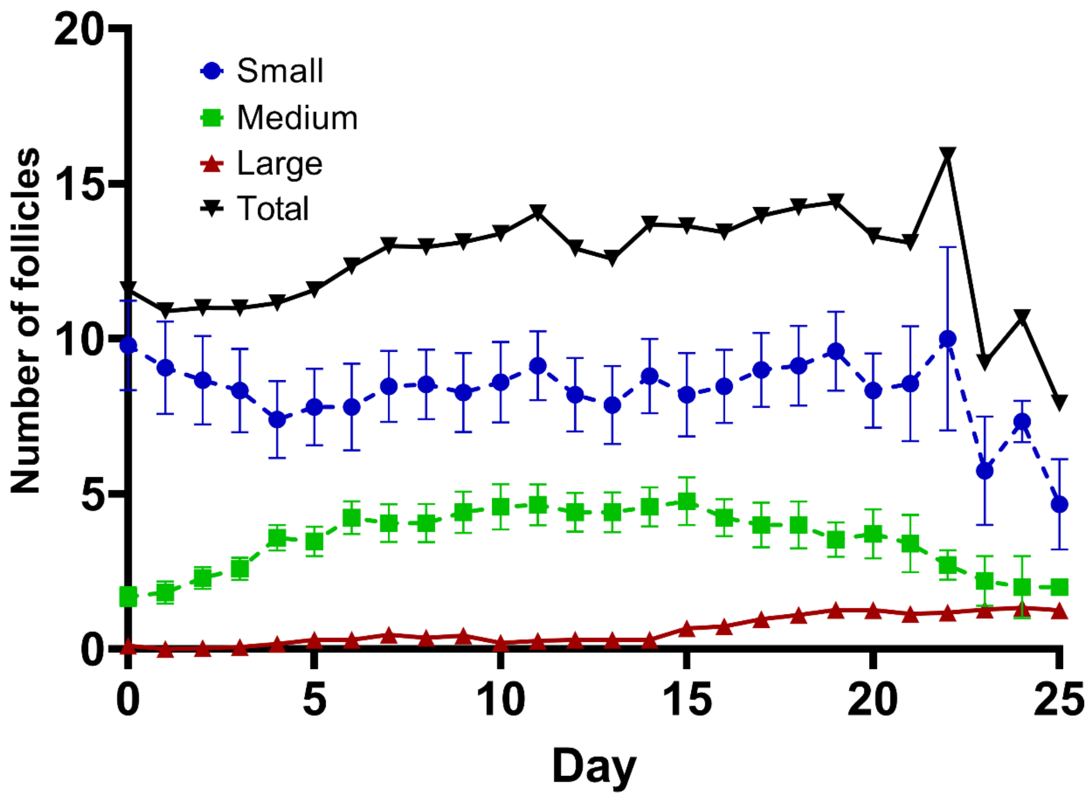

Figure S2. Number of small (≥5≤10 mm diameter); medium (11-19 mm diameter) and large (≥ 20 mm diameter) follicles during four estrous cycles of eight Caribbean jennies in the tropics.

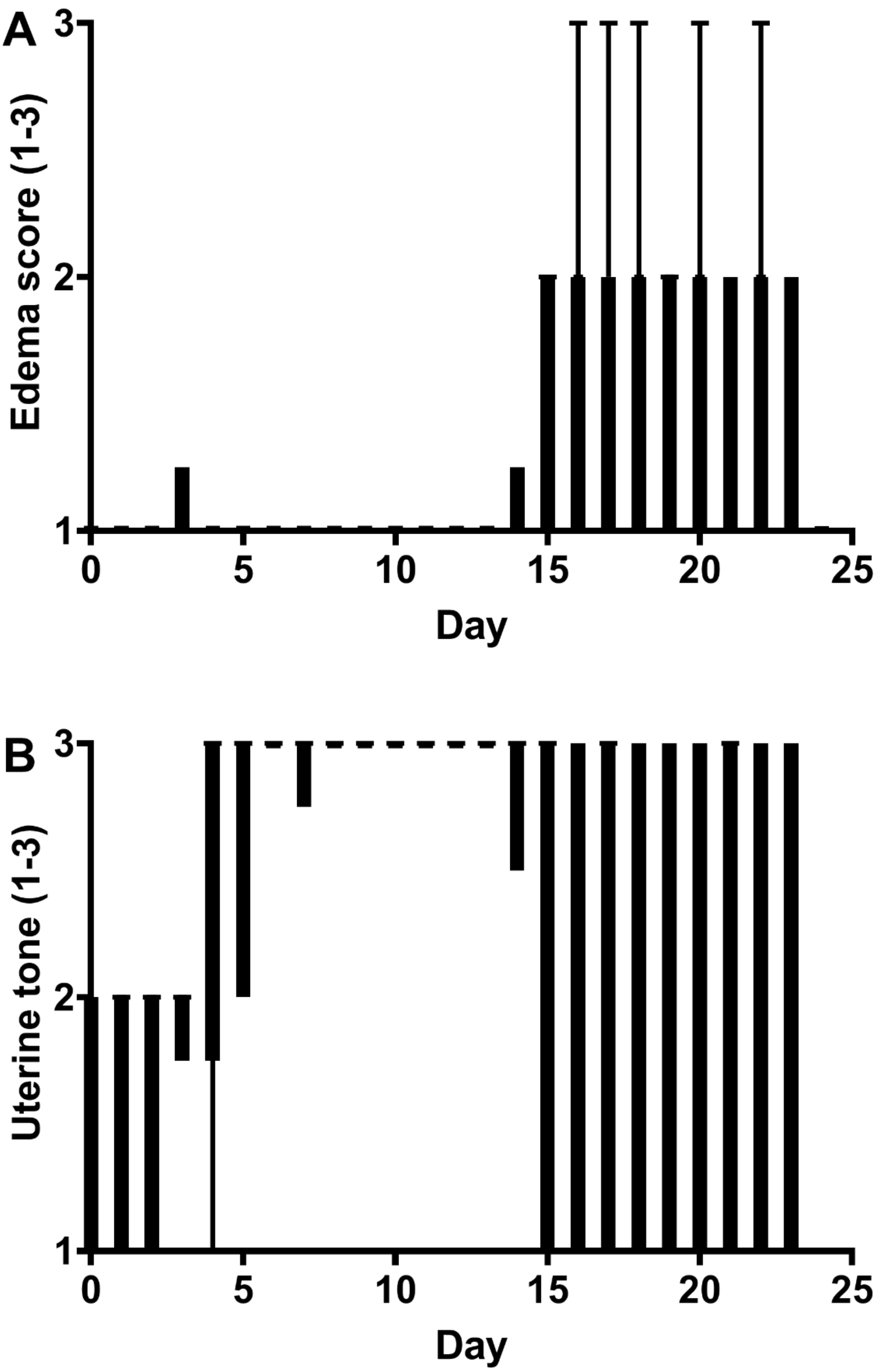

Figure S3. Box plot of (A) endometrial edema (B) and uterine tone scores of eight Caribbean jennies during four consecutive estrous cycles in the Tropics. Uterine tone (1 to 3; 1: flaccid and 3: turgid) and endometrial edema (1: minimal edema to 4: exacerbated edema) scores were evaluated by transrectal digital palpation and ultrasonography, respectively.
